# Is individual consistency in body mass and reproductive decisions linked to individual specialization in foraging behavior in a long‐lived seabird?

**DOI:** 10.1002/ece3.2213

**Published:** 2016-06-08

**Authors:** Nina Dehnhard, Marcel Eens, Nicolas Sturaro, Gilles Lepoint, Laurent Demongin, Petra Quillfeldt, Maud Poisbleau

**Affiliations:** ^1^ Department Biology – Behavioural Ecology & Ecophysiology GroupUniversity of AntwerpCampus Drie Eiken, Universiteitsplein 12610Antwerp (Wilrijk)Belgium; ^2^ Department of Migration and Immuno‐EcologyMax Planck Institute for OrnithologyAm Obstberg 178315RadolfzellGermany; ^3^ Department of BiologyUniversity of Konstanz78457KonstanzGermany; ^4^ Laboratory of OceanologyMARE CentreUniversity of LiègeB6C, Sart Tilman4000LiègeBelgium; ^5^ Department of Animal Ecology & SystematicsJustus‐Liebig University GießenHeinrich‐Buff‐Ring 38D‐35392GießenGermany

**Keywords:** Clutch mass, environmental variability, *Eudyptes chrysocome*, global climate change, phenology, sea surface temperature, Southern Annular Mode, Southern Oscillation Index, southern rockhopper penguin, stable isotopes

## Abstract

Individual specialization in diet or foraging behavior within apparently generalist populations has been described for many species, especially in polar and temperate marine environments, where resource distribution is relatively predictable. It is unclear, however, whether and how increased environmental variability – and thus reduced predictability of resources – due to global climate change will affect individual specialization. We determined the within‐ and among‐individual components of the trophic niche and the within‐individual repeatability of δ^13^C and δ^15^N in feathers and red blood cells of individual female southern rockhopper penguins (*Eudyptes chrysocome*) across 7 years. We also investigated the effect of environmental variables (Southern Annular Mode, Southern Oscillation Index, and local sea surface temperature anomaly) on the isotopic values, as well as the link between stable isotopes and female body mass, clutch initiation dates, and total clutch mass. We observed consistent red blood cell δ^13^C and δ^15^N values within individuals among years, suggesting a moderate degree of within‐individual specialization in C and N during the prebreeding period. However, the total niche width was reduced and individual specialization not present during the premolt period. Despite significant interannual differences in isotope values of C and N and environmental conditions, none of the environmental variables were linked to stable isotope values and thus able to explain phenotypic plasticity. Furthermore, neither the within‐individual nor among‐individual effects of stable isotopes were found to be related to female body mass, clutch initiation date, or total clutch mass. In conclusion, our results emphasize that the degree of specialization within generalist populations can vary over the course of 1 year, even when being consistent within the same season across years. We were unable to confirm that environmental variability counteracts individual specialization in foraging behavior, as phenotypic plasticity in δ^13^C and δ^15^N was not linked to any of the environmental variables studied.

## Introduction

There is increasing evidence for individual specialization in resource use within generalist species. In such cases, the niche of the specialized individuals is substantially smaller than that of the population as a whole (Roughgarden 1972; Bolnick et al. 2003). Examples of specialization are especially prevalent in the foraging behavior and diet of marine predators (Bolnick et al. 2003). According to optimal foraging theory, the level of individual specialization depends on the abundance and diversity of resources, as well as on individuals' phenotypic traits: Reduced availability of preferred resources will lead to increased intraspecific competition and an expansion of the individual's niche to include less valuable resources. However, it depends on the individuals' preferences for different resources whether intraspecific competition will increase or decrease individual specialization (Araújo et al. 2011). Individuals may on the other hand also differ in their optimal diet, depending, for example, on their ability to detect, capture, and handle prey, the risk of predation involved in capturing a specific prey, physiological needs for specific nutrients, or differences in their boldness/shyness (Schoener 1971; Araújo et al. 2011; Patrick and Weimerskirch 2014).

Differences in intrinsic quality may also explain the link between individual specialization in resource use and differential investment in reproduction: A number of studies have shown a link between individual specialization in either foraging behavior or diet and measures of reproductive success (e.g., Annett and Pierotti 1999; Patrick and Weimerskirch 2014) or timing of reproduction (Ducatez et al. 2008; Anderson et al. 2009). On the other hand, under fluctuating prey resources, individual specialization may only be beneficial over shorter time periods, with advantages leveling off in the long‐term (e.g., Woo et al. 2008; van de Pol et al. 2010). The occurrence of individual specialization and its long‐term benefits may thus also depend on the predictability of resources, which should decline over temporal and spatial scales. Indeed, foraging site fidelity decreased with foraging range in temperate and polar seabird species, which inhabit biomes with relatively predictable resource patches (Weimerskirch 2007). In contrast, no such relationship was found for tropical seabirds that forage in less predictable waters (Weimerskirch 2007).

If individual specialization depends on predictability of the habitat/environment (also see Wakefield et al. 2015), this raises the question whether long‐term individual specialization will decrease with the increased environmental variability caused by global climate change. To cope with the manifold effects of global climate change, phenotypic plasticity is emphasized as a critical characteristic especially for long‐lived species (Vedder et al. 2013). Phenotypic plasticity is the ability of the genotype to modify its phenotype (Houston and McNamara 1992), for example, when an individual modifies its foraging behavior or breeding behavior. Notably, phenotypic plasticity and individual specialization in behavioral responses *per se* do not contradict each other. At least as long as all individuals show the same level of phenotypic plasticity, individual differences in behavior (and therefore the degree of individual specialization) remain consistent (cf. fig. 1b in Nussey et al. 2005a). Such differential consistency is also referred to as “broad‐sense repeatability” (Stamps and Groothuis 2010). However, the level of phenotypic plasticity may also differ among individuals, with some reacting more plastically than others. This would counteract consistent among‐individual differences in behavior (cf. fig. 1c in Nussey et al. 2005a) and therefore the repeatability of individuals' behavioral responses. In fact, among‐individual differences in phenotypic plasticity for a trait may enhance the speed of micro‐evolutionary adaptation (Dingemanse and Wolf 2013), and may be critically important for animals to adapt to changes in their environment. This is because such among‐individual differences in phenotypic plasticity could increase the lifetime reproductive success of the better adapted and more plastic individuals (Nussey et al. 2005b, 2007; Gienapp et al. 2008).

Stable isotope analysis presents a minimally invasive method to study the approximate foraging area and resource use and identify specialist and generalist patterns both within and among species (Bearhop et al. 2004; Kowalczyk et al. 2014; Polito et al. 2015). Furthermore, stable isotope analysis can be applied during migration and/or wintering periods when the deployment of GPS devices or a more direct assessment of diet is difficult to impossible (Cherel et al. 2007; Hinke et al. 2015). The carbon stable isotope ratio (^13^C/^12^C, hereafter δ^13^C) mainly varies spatially within the marine ecosystem, with distance from land and on a gradient from benthic to pelagic food webs (reviewed in Rubenstein and Hobson 2004) and according to latitude (Cherel and Hobson 2007). Therefore, δ^13^C serves as an indicator of the foraging area of an animal (Cherel and Hobson 2007). In contrast, ^15^N accumulates stepwise from diet to consumer tissues, and the nitrogen isotopic ratio (^15^N/^14^N, hereafter δ^15^N) therefore indicates the trophic level of an animal within the food web (Minagawa and Wada 1984).

**Figure 1:**
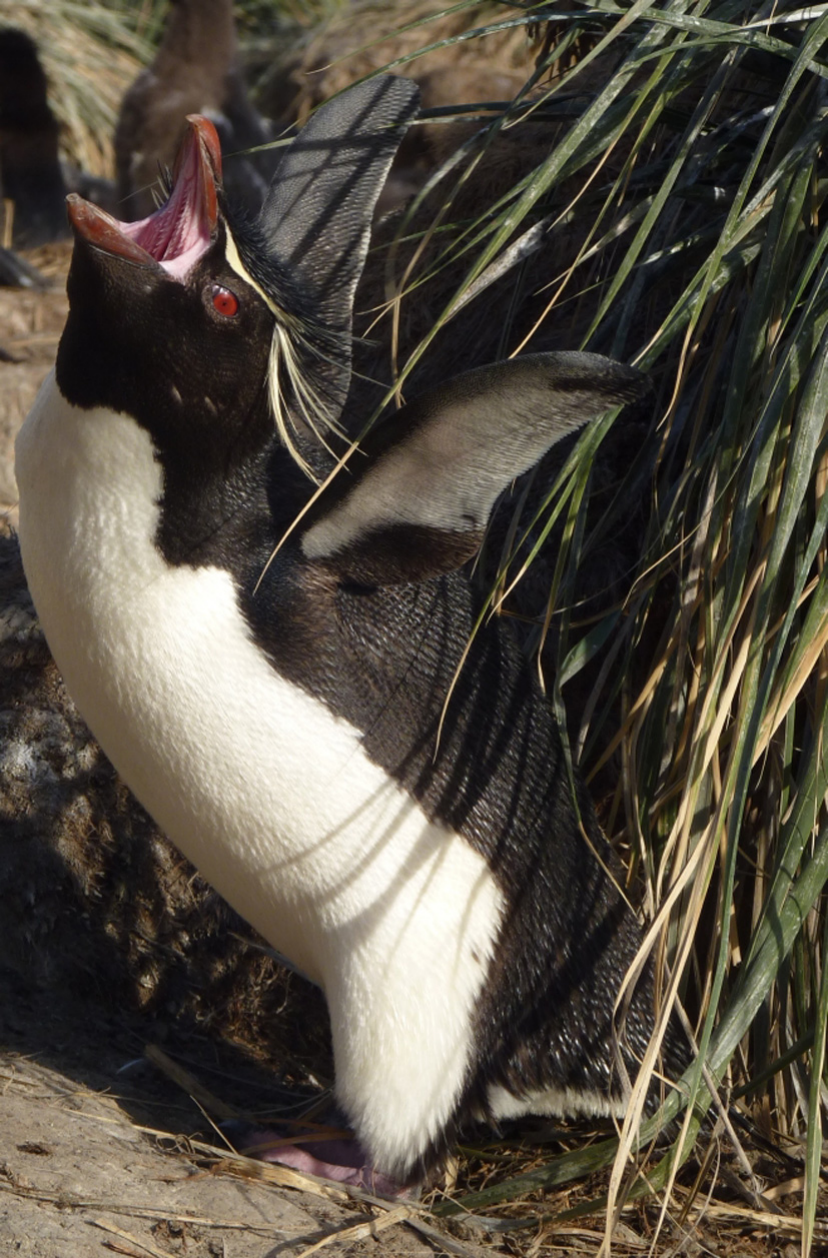
Displaying female southern rockhopper penguin (*Eudyptes chryosocome chrysocome*).

We investigated the degree of individual isotopic specialization, reflecting the individuals' foraging behavior (and therefore specifically the trophic level of prey taken and the utilized foraging areas) across several years, using stable isotope analysis applied to tissues reflecting the prebreeding and the premolt periods in female southern rockhopper penguins (*Eudyptes chrysocome chrysocome*; hereafter SRP; Fig. 1). This species is ideal to study individual specialization in foraging behavior over several years as SRP are long‐lived and highly philopatric to their nest sites (Dehnhard et al. 2013b). Within their distribution area, δ^13^C varies on the latitudinal and longitudinal scale (Quillfeldt et al. 2010), implying that information on δ^13^C can be used to infer approximate foraging areas (Dehnhard et al. 2011). Furthermore, being located relatively low in the local food web (Weiss et al. 2009), while feeding on a broad range of fish, squid, and crustacean species (i.e., being food generalists; Pütz et al. 2013), SRP appear to be sensitive to environmentally driven changes in the food web. Previous studies have shown that body mass, egg masses, and timing of breeding in this species are linked to the prebreeding environmental conditions (Dehnhard et al. 2015a,b). At the same time, adults are highly consistent in their body mass at commencement of breeding and in their investment into egg masses across years (Dehnhard et al. 2015a). Long‐term specialization of individuals on specific prebreeding foraging areas or food items might be an explanation for these findings. However, to the best of our knowledge, no study so far has investigated the level of individual specialization and potential ecological consequences for these penguins.

The aims of this study were to investigate: (1) the degree of individual specialization by comparing the within‐ and among‐individual variation in the total isotopic niche width; (2) the broad‐sense repeatability (individual consistency) in foraging behavior (i.e., trophic level of prey and utilized foraging areas) using δ^15^N and δ^13^C; (3) the level of phenotypic plasticity in trophic level and foraging area within individuals in response to several candidate environmental variables and to test whether it differs among individuals (this would counteract individual specialization but could enhance the species' speed of adaptation to changes in the environment); and (4) whether within‐ and among‐individual differences in isotopic compositions are linked to body mass, clutch initiation date, and clutch mass (i.e., whether specialization in foraging behavior affects breeding parameters).

## Materials and Methods

### Study area and field methods

Fieldwork was conducted in the “Settlement Colony” on New Island in the Falkland Islands/Islas Malvinas (51°43′S, 61°17′W) between October 2006 and December 2013. In the framework of an ongoing project on maternal investment starting in 2006, we gradually marked 461 randomly chosen adult females with passive integrated transponders (PITs; 23‐mm‐long, glass‐encapsulated, TIRIS, Texas Instruments, USA; see Dehnhard et al. 2013a for more details). Each year (except in 2011 when no fieldwork was conducted), we collected data on egg laying dates, egg masses, and female body masses. We also collected blood and feather samples from the same individuals (except in 2009 when no feather samples were collected). The sex of the birds was determined from a combination of morphological and behavioral observations (Poisbleau et al. 2010); males are larger than females and both sexes have a fixed pattern of nest attendance and incubation shifts which hardly varies among years (Strange 1982). Briefly, after their winter migration, males arrive in breeding colonies in the first week of October, followed by the females a few days later. Both males and females stay ashore and fast during the entire courtship and egg laying period and the first incubation shift. In the middle of November, males leave colonies for a ca. 10‐day foraging trip, while females incubate eggs alone during this second incubation shift. Females leave the colony for foraging only after the males have returned. Molt occurs in late March or April, allowing for a foraging trip of at least 3 weeks after chicks have fledged (Strange 1982). Like all penguins, SRP molt their entire plumage simultaneously and fast on land during this time (Pütz et al. 2013).

We visited the colony daily from mid‐October onward to follow focal females equipped with a transponder and record individual clutch initiation dates (corresponding to the A‐egg laying dates). We weighed both A and B eggs to the closest 0.1 g using a digital balance on the day when they were first observed. Total clutch mass was calculated as the sum of A‐egg mass and B‐egg mass.

We captured females on their clutch initiation dates on their nests, covered their head to minimize stress, and took a small blood sample (<1 mL) from the brachial vein, using a heparinized syringe and a 23‐G needle. Although sodium heparin contains carbon, previous studies in other vertebrates could not find significant effects of this anticoagulant on red blood cell stable isotope measurements (Kim and Koch 2012; Lemons et al. 2012), and we therefore assumed that using heparinized syringes did not affect the measured isotopic values in red blood cells.

Feathers were gently pulled out of the skin (2 white feathers per individual). Birds were then weighed to the nearest 20 g with an electronic balance following Poisbleau et al. (2010). Capture and handling did not exceed 10 min, and birds were released a few meters away from their nests and returned to their partner on the nest. For logistical reasons, some females were captured before or after their clutch initiation date, and we applied corrections for these cases (see Supplement 1).

### Stable isotope analyses

Blood samples were stored on ice while being in the field and subsequently centrifuged. Plasma was removed and red blood cell samples were frozen (−20°C) and later dried in a drying furnace (at 60°C) or lyophilized. Dried red blood cells were ground to a fine powder and homogenized. Aliquots of 0.80 to 0.95 mg were weighed into tin cups. Using one feather per individual bird, we excluded calamus and rachis and cut the rest of the feather material into small pieces (using stainless steel scissors) which was then all filled into a tin cup, resulting in aliquots of 0.8 to 1.3 mg.

Stable isotope analyses of carbon and nitrogen were conducted at the Laboratory of Oceanology, MARE Centre at the University of Liège as described in Thiebot et al. (2015). Analytical precision (± SD) on replicated samples equaled ± 0.3 and ± 0.5‰ for δ^13^C and δ^15^N, respectively.

### Environmental variables

We evaluated the effect of three different environmental variables on isotope values: the two broad‐scale climatic indices Southern Annular Mode (SAM) and Southern Oscillation Index (SOI) as well as local sea surface temperature anomaly (SSTA). All three variables are temperature‐related, and we here also consider them as potential proxies for food availability. A direct quantification of food availability in the ocean is nearly impossible. Ocean temperatures, however, are closely linked to primary productivity and therefore food availability. For example, areas of upwelling, where nutrient‐rich water from the ocean's bottom is breaching the surface, are characterized by low water temperatures and high primary productivity (Mann and Lazier 2006). On the other hand, the water column undergoes a shallower and more stable stratification under higher temperatures, resulting in a reduced availability of macronutrients for primary producers in the light‐exposed upper zone of the ocean (Behrenfeld et al. 2006). As a consequence, ocean productivity decreases (Behrenfeld et al. 2006) and changes in the composition of the food web occur under increased ocean temperatures (Moline et al. 2004). Temperature changes can therefore affect ocean productivity and consequently availability of food in space and time (Durant et al. 2007, 2010).

SAM is the dominant mode of atmospheric variability in the Southern Hemisphere, with distinct effects on wind patterns and sea surface temperatures (Marshall 2003). SOI (also referred to as El Niño Southern Oscillation or ENSO) is defined as the air‐pressure difference between the mid‐Pacific (Tahiti) and West Pacific (Darwin). Both of these broad‐scale climatic indices have effects on sea surface temperatures in the South Atlantic Ocean, with positive SAM and SOI indices coupled to lower surface temperatures (Kwok and Comiso 2002; Meredith et al. 2008). Local SSTA represent a different spatial scale and thus reflect environmental conditions close to the colony. Including environmental variables that reflect not only local conditions at the breeding sites but also over a wider spatial scale is important in the case of our study as SRP are migratory and may therefore not be able to detect local conditions until shortly before their arrival at their breeding colonies (c.f. Frederiksen et al. 2004). All three variables have previously been shown to affect either breeding biology or population dynamics of other seabird species, including SRP (Frederiksen et al. 2007; Emmerson et al. 2011; Baylis et al. 2012; Hindell et al. 2012; Dehnhard et al. 2013b, 2015b).

To examine the effect of environmental variables on red blood cell isotope compositions (i.e., reflecting the prebreeding period), we proceeded similarly to Lynch et al. (2012) and averaged environmental variables from August and September. For the premolt period (i.e., isotopic compositions from feathers), we averaged environmental variables from February and March. We chose these time periods based on the breeding and molting scheme of SRP (Strange 1982), the estimated turnover time of red blood cell isotopes (see Thiebot et al. 2015), and the accumulation of body reserves prior to molting (Green et al. 2009). We did not consider a time lag between environmental variables and their potential effects as: (1) SRP are feeding at low trophic level prey that should be affected by environmental changes rapidly; and (2) previous studies found immediate effects (i.e., without a time lag) of environmental conditions on SRP female body masses, egg masses, and egg laying dates (Dehnhard et al. 2015a,b).

Monthly SAM and SOI were downloaded from the British Antarctic Survey (http://www.nerc-bas.ac.uk/icd/gjma/sam.html) and the University Center for Atmospheric Research Climate Analysis Section Data Catalogue (http://www.cgd.ucar.edu/cas/catalog/climind/SOI.signal.ascii), respectively. For local SSTA (in °C), we selected a 2° grid in the west of New Island (50–52°S, 61–63°W). This area is known to be the major foraging location of SRP during the breeding season (Ludynia et al. 2012, 2013) and may also be used by the penguins shortly before arrival to the breeding sites in spring. Monthly SSTA were based on the difference between monthly sea surface temperature and the long‐term monthly average (from 1971 to 2000) and were obtained from the National Oceanic and Atmospheric Administration (http://iridl.ldeo.columbia.edu/SOURCES/.NOAA/.NCEP/.EMC/.CMB/.GLOBAL/.Reyn_SmithOIv2/.monthly/).

### Statistical analyses

We considered only females for which we had obtained blood as well as body mass, A‐egg mass and B‐egg mass for at least 3 years, and feather samples for at least 2 years. This resulted in a database of 130 records (between 11 and 25 per year) from 30 different females. Over the 7 years, individuals were blood‐sampled on average in 4.3 ± 1.0 SD (min. 3, max. 6) years. As feathers were not sampled in 2009, the corresponding number for feather samples was lower (average 3.67 ± 1.1 SD; min. 2, max. 5).

To determine the degree of individual specialization within our study population, we followed the approach of Bolnick et al. (2003) and distinguished between the within‐individual component (WIC) and the among‐individual component (AIC) of the population's total niche width (TNW). We adapted the approach by Jaeger et al. (2009) and – separately for δ^15^N and δ^13^C and both analyzed tissues – calculated the WIC as the average of the isotopic variance within all samples obtained per individual across the study period of 7 years. For the AIC, we calculated the average isotopic value per individual bird across the 7 years and then determined the variance between the averaged values per individual. Generalist populations are characterized by a large TNW. Considering that WIC + AIC = TNW, a generalist population with a high degree of individual specialization is characterized by a large AIC, such that the WIC/TNW ratio is decreasing with increasing individual specialization (Roughgarden 1972; Bolnick et al. 2003). Notably, the WIC/TNW ratio forms a continuum, and while the upper limit of WIC/TNW = 1 is well defined as a true generalist population, drawing a lower limit for a generalist population (or, in other words, an upper limit for what can still be called individual specialization) is more difficult. In a recent review of the existing literature on individual specialization across taxa, the average WIC/TNW ratio was 0.66 (and therefore closer to 1 than to 0), although the vast majority of the included studies documented individual specialization (Araújo et al. 2011). As such, even a WIC/TNW of 0.7 might still be referred to as “moderate specialization” (Jaeger et al. 2010) as this would reflect that the individuals' niche is only 70% as broad as the niche of the entire population.

To determine individual consistency in foraging behavior, we calculated the among‐year repeatabilities for red blood cell and feather δ^15^N and δ^13^C within females, using REML‐based linear mixed models as described in Nakagawa and Schielzeth (2010), in the rptR package (Schielzeth and Nakagawa 2013) in R (version 3.1.1; R Core Team 2014).

We then tested the effect of year and the influence of environmental variables on red blood cell and feather δ^15^N and δ^13^C by fitting linear mixed effects models (first set of LMM). We also used LMM to test the interplay between δ^15^N and δ^13^C and female body mass, clutch initiation date, and total clutch mass (second set of LMM). In order to test for individual‐level plasticity, we used within‐individual‐centered data as explanatory variables to differentiate within‐individual‐level responses from among‐individual‐level responses and also tested for the support of individual random slopes, as previously described by van de Pol and Wright (2009). We therefore calculated within‐individual‐centered (*x*_ij_−${\bar{x}}_{j}$) SAM, SOI, and SSTA for the first set of LMM (to investigate the effect of environmental variables on red blood cell and feather δ^15^N and δ^13^C) and within‐individual‐centered δ^15^N and δ^13^C for the second set of LMM (relationship between stable isotopes and female body mass, clutch initiation date, and total clutch mass). Briefly, *x*_ij_ would, for example, reflect the SAM experienced by individual j in year i. ${\bar{x}}_{j}$ would then be the average SAM experienced by individual j across all years that individual j was included in the study (e.g., ${\bar{x}}_{j}$ would be calculated as the average SAM in the years 2006, 2007, and 2008 for an individual that was blood‐sampled in these 3 years). In the models, (*x*_ij_−${\bar{x}}_{j}$) would consequently reflect within‐individual effects and (${\bar{x}}_{j}$) would reflect among‐individual effects.

For the first set of LMM, we conducted separate models for all four dependent variables, namely red blood cell and feather δ^15^N and δ^13^C. We first tested for the effect of year (explanatory variable) on these dependent variables, controlling for the repeated sampling of the same females by including bird identity as a random effect. Thereafter, we continued with the three (individual‐centered) candidate environmental variables (SAM, SOI, and SSTA). As not only linear, but also quadratic effects could be possible (Cimino et al. 2014), we also included the quadratic terms of the environmental variables in models. As environmental variables were partly correlated with each other during the study period (e.g., February–March SOI and SSTA: Pearson's *r* = 0.85, *P* = 0.015, *N* = 7 years), we decided against fitting several (or all) explanatory variables into one model but instead – in order to avoid collinearity and retain the same procedure for all models – ran one model per explanatory variable – separately for all dependent variables (i.e., a total of 28 models, including null models without any explanatory variable). Each of these models contained bird identity and year as independent random effects. After identifying the best fixed effects model structure (i.e., the best explanatory environmental variable), we validated whether the within‐individual effect was significant. Only if this was the case (see van de Pol and Wright 2009), we continued to validate the random‐effect model structure by testing whether individual random slopes were supported in the model or not.

We proceeded similarly for the second set of LMM. Here, we conducted separate models for the effect of red blood cell δ^15^N and δ^13^C on the three dependent variables female body mass, clutch initiation date, and total clutch mass. To account for differences among years in both stable isotope variables (see Results) as well as female body mass, clutch initiation date, and total clutch mass (Dehnhard et al. 2015a,b), we standardized all of these values within each year, using *z*‐scores. We then centered red blood cell δ^15^N and δ^13^C individually and included these individual‐centered values as explanatory variables in separate models (as δ^15^N and δ^13^C were significantly correlated; Pearson's *r* = 0.53, *P* < 0.001), resulting in a total of 9 models including null models. Again, we included bird identity and year as independent random effects, and, only if the within‐individual effect was significant, tested for the support of individual random slopes in the model.

All models were fit with the lme4 package (Bates et al. 2011) in R and based on restricted maximum likelihood (REML). *P*‐values were obtained from likelihood‐ratio tests (fit with maximum likelihood) based on the model with and without the concerned variable. We followed Nakagawa and Schielzeth (2013) to calculate marginal *R* ^2^ values ($R_{m}^{2}$, for the variance explained only by fixed effects) and conditional *R* ^2^ values ($R_{c}^{2}$, based on the variance explained by both fixed and random effects). Models were validated using the protocols described in Zuur et al. (2009).

## Results

### Total niche width (TNW) and degree of individual specialization

**Figure 2:**
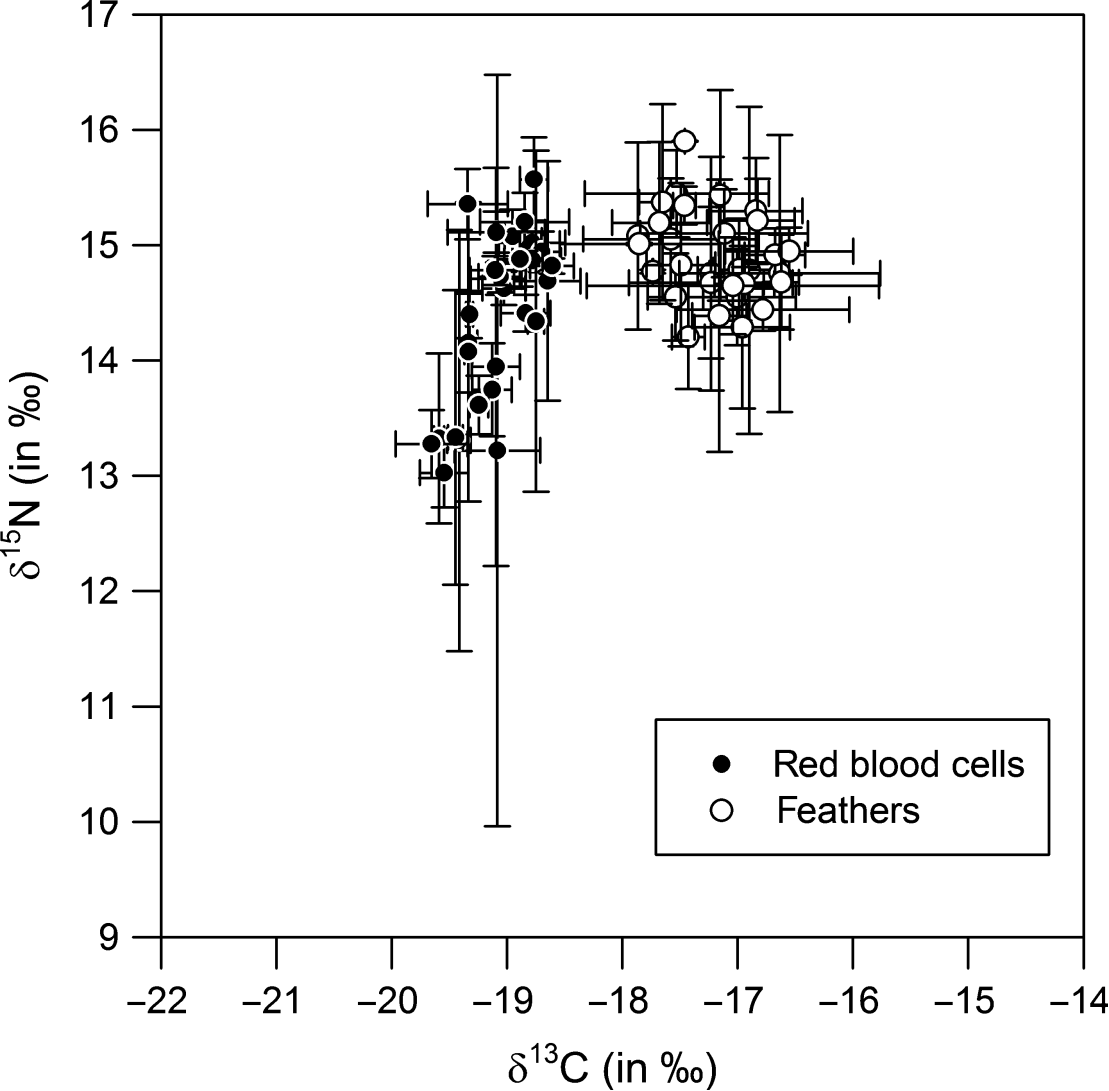
Within‐ and among‐individual variation in red blood cell and feather δ^13^C and δ^15^N of southern rockhopper penguins. Circles mark the average value per individual, and error bars show the within‐individual variance among years. The variance among the average values per individual (circles) is defined as the among‐individual component (AIC) of the trophic niche. The average value of the within‐individual variance (error bars) presents the within‐individual component (WIC) of the trophic niche.

During the prebreeding period (as reflected by red blood cell isotopes), SRP females showed a wider TNW for δ^15^N than for δ^13^C (Table 1, Fig. 2). For δ^15^N, within‐ and among‐individual variation during the 7‐year study period was similar (Table 1, Fig. 2) and the WIC/TNW was 0.52. For δ^13^C, among‐individual variation was lower than within‐individual variation, resulting in a WIC/TNW ratio of 0.68 (Table 1).

**Table 1:** Total niche width (TNW), within‐individual and among‐individual components (WIC and AIC), and the WIC/TNW ratio reflecting the degree of individual specialization, calculated from red blood cells and feathers of the same individual females across several years. WIC reflects the variation within individuals and AIC the variation among individuals. WIC/TNW ranges from 0 to 1, with increasing individual specialization as values approach 0. *N* = 130 red blood cell and *N* = 110 feather samples originating from 30 individual female rockhopper penguins sampled three to six times (red blood cells), and two to five times (feathers), respectively, across 7 years

	Red blood cells		Feathers	
	δ^15^N	δ^13^C	δ^15^N	δ^13^C
TNW (‰)	1.12	0.28	0.67	0.54
WIC (‰)	0.59	0.19	0.53	0.39
AIC (‰)	0.54	0.09	0.15	0.15
WIC/TNW	0.52	0.68	0.78	0.72

Feather stable isotope data, reflecting the foraging behavior during the premolt period, showed a similar TNW for both δ^15^N and δ^13^C (Table 1, Fig. 2). The among‐individual variation was markedly smaller than the within‐individual variation, resulting in a WIC/TNW ratio of 0.78 and 0.72, respectively (Table 1).

### Repeatability as a measure of individual consistency

Red blood cell δ^15^N and δ^13^C were significantly repeatable within individual females across years (Table 2). In contrast, feather δ^15^N and δ^13^C were not repeatable within individuals, reflecting no such individual consistency for the premolt period. There was no significant correlation in either δ^15^N or δ^13^C between red blood cells and feathers (Pearson's *r* ≤ |0.16|, *P* ≥ 0.101).

**Table 2:** Repeatability of red blood cell and feather isotope ratios within individual females across multiple years. For sample sizes, see Table 1

	Repeatability±SE	*P*‐value
Red blood cells		
δ^15^N	0.472 ± 0.086	<0.001
δ^13^C	0.203 ± 0.101	0.037
Feathers		
δ^15^N	0.013 ± 0.065	1.000
δ^13^C	0.025 ± 0.055	1.000

### Variability of stable isotope compositions among years and with environmental variables

**Figure 3:**
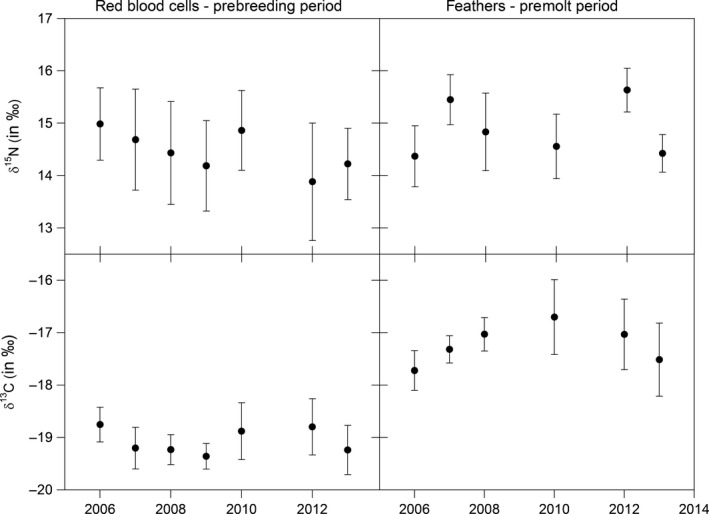
Annual variation in red blood cell (left column) and feather (right column) δ^15^N and δ^13^C (means ± SD). Note that no red blood cell samples were collected in 2011 and no feather samples in 2009 and 2011.

Red blood cell δ^15^N and δ^13^C differed significantly among years (Fig. 3; *F*_6_ = 7.55 and 9.58, respectively, both *P* < 0.001), and the same was true for feather δ^15^N and δ^13^C (Fig 2; *F*_5_ = 20.05 and 8.41, respectively, both *P* < 0.001). Nevertheless, none of the models with environmental variables to account for annual variability in red blood cell and feather δ^15^N and δ^13^C performed substantially better than the null model (Table 3). The within‐individual effect was not significant in any of the models (all *F*_1_ < 3.24, *P* ≥ 0.066), and it was, for this reason, not indicated to include individual random slopes to test for among‐individual differences in phenotypic plasticity into models. Overall, we therefore found no evidence for phenotypic plasticity in isotopic composition in response to the chosen environmental variables.

**Table 3:** Comparison of linear mixed effects models for red blood cell and feather δ^15^N and δ^13^C. Models contained environmental variables (SSTA, sea surface temperature anomaly; SAM, Southern Annular Mode; SOI, Southern Oscillation Index) as explanatory variables, with both the within‐individual‐centered data point (*x*_ij_−${\bar{x}}_{j}$) as well as the average value for each individual across years (${\bar{x}}_{j}$). Environmental variables were averaged for the months of August and September for models on red blood cells isotopes, and for the months of February and March for those on feather isotopes. We also included null models (without any environmental variable) for comparison in the modeling process. All models (including null models) contained bird identity and year as independent random effects. AIC presents the Akaike information criterion. Marginal *R* ^2^ values ($R_{m}^{2}$) denote the variance explained only by fixed effects, whereas conditional *R* ^2^ values ($R_{c}^{2}$) express the variance explained by both fixed and random effects

	AIC	ΔAIC	$R_{m}^{2}$	$R_{c}^{2}$
Red blood cell δ^15^N				
Null	304.907	0.000	0.000	0.625
SSTA (*x*_ij_−${\bar{x}}_{j}$)+SSTA (${\bar{x}}_{j}$)	305.754	0.847	0.039	0.636
SOI (*x*_ij_−${\bar{x}}_{j}$)+SOI (${\bar{x}}_{j}$)	307.973	3.066	0.015	0.645
SAM (*x*_ij_−${\bar{x}}_{j}$)+SAM (${\bar{x}}_{j}$)+(SAM (*x*_ij_−${\bar{x}}_{j}$))^2^+(SAM (${\bar{x}}_{j}$))^2^	308.555	3.648	0.051	0.645
SAM (*x*_ij_−${\bar{x}}_{j}$)+SAM (${\bar{x}}_{j}$)	308.879	3.972	0.001	0.645
SSTA (*x*_ij_−${\bar{x}}_{j}$)+SSTA (${\bar{x}}_{j}$)+(SSTA (*x*_ij_−${\bar{x}}_{j}$))^2^+(SSTA (${\bar{x}}_{j}$))^2^	309.325	4.418	0.048	0.652
SOI (*x*_ij_−${\bar{x}}_{j}$)+SOI (${\bar{x}}_{j}$)+(SOI (*x*_ij_−${\bar{x}}_{j}$))^2^+(SOI (${\bar{x}}_{j}$))^2^	311.502	6.596	0.015	0.653
Red blood cell δ^13^C				
Null	150.788	0.000	0.000	0.504
SSTA (*x*_ij_−${\bar{x}}_{j}$)+SSTA (${\bar{x}}_{j}$)	150.931	0.142	0.072	0.512
SAM (*x*_ij_−${\bar{x}}_{j}$)+SAM (${\bar{x}}_{j}$)	153.383	2.594	0.044	0.528
SOI (*x*_ij_−${\bar{x}}_{j}$)+SOI (${\bar{x}}_{j}$)	154.174	3.385	0.007	0.538
SSTA (*x*_ij_−${\bar{x}}_{j}$)+SSTA (${\bar{x}}_{j}$)+(SSTA (*x*_ij_−${\bar{x}}_{j}$))^2^+(SSTA (${\bar{x}}_{j}$))^2^	154.471	3.682	0.069	0.535
SAM (*x*_ij_−${\bar{x}}_{j}$)+SAM (${\bar{x}}_{j}$)+(SAM (*x*_ij_−${\bar{x}}_{j}$))^2^+(SAM (${\bar{x}}_{j}$))^2^	155.321	4.532	0.063	0.538
SOI (*x*_ij_−${\bar{x}}_{j}$)+SOI (${\bar{x}}_{j}$)+(SOI (*x*_ij_−${\bar{x}}_{j}$))^2^+(SOI (${\bar{x}}_{j}$))^2^	156.784	5.996	0.016	0.510
Feather δ^15^N				
SOI (*x*_ij_−${\bar{x}}_{j}$)+SOI (${\bar{x}}_{j}$)+(SOI (*x*_ij_−${\bar{x}}_{j}$))^2^+(SOI (${\bar{x}}_{j}$))^2^	198.618	0.000	0.058	0.624
Null	199.867	1.249	0.000	0.564
SSTA (*x*_ij_−${\bar{x}}_{j}$)+SSTA (${\bar{x}}_{j}$)+(SSTA (*x*_ij_−${\bar{x}}_{j}$))^2^+(SSTA (${\bar{x}}_{j}$))^2^	201.183	2.565	0.058	0.607
SSTA (*x*_ij_−${\bar{x}}_{j}$)+SSTA (${\bar{x}}_{j}$)	202.576	3.957	0.022	0.601
SOI (*x*_ij_−${\bar{x}}_{j}$)+SOI (${\bar{x}}_{j}$)	203.083	4.465	0.004	0.610
SAM (*x*_ij_−${\bar{x}}_{j}$)+SAM (${\bar{x}}_{j}$)	203.486	4.868	0.024	0.615
SAM (*x*_ij_−${\bar{x}}_{j}$)+SAM (${\bar{x}}_{j}$)+(SAM (*x*_ij_−${\bar{x}}_{j}$))^2^+(SAM (${\bar{x}}_{j}$))^2^	204.926	6.307	0.040	0.612
Feather δ^13^C				
Null	210.205	0.000	0.000	0.369
SOI (*x*_ij_−${\bar{x}}_{j}$)+SOI (${\bar{x}}_{j}$)	210.316	0.111	0.097	0.385
SOI (*x*_ij_−${\bar{x}}_{j}$)+SOI (${\bar{x}}_{j}$)+(SOI (*x*_ij_−${\bar{x}}_{j}$))^2^+(SOI (${\bar{x}}_{j}$))^2^	211.483	1.278	0.091	0.374
SSTA (*x*_ij_−${\bar{x}}_{j}$)+SSTA (${\bar{x}}_{j}$)	211.990	1.786	0.118	0.394
SAM (*x*_ij_−${\bar{x}}_{j}$)+SAM (${\bar{x}}_{j}$)	212.486	2.281	0.061	0.395
SSTA (*x*_ij_−${\bar{x}}_{j}$)+SSTA (${\bar{x}}_{j}$)+(SSTA (*x*_ij_−${\bar{x}}_{j}$))^2^+(SSTA (${\bar{x}}_{j}$))^2^	212.673	2.469	0.084	0.401
SAM (*x*_ij_−${\bar{x}}_{j}$)+SAM (${\bar{x}}_{j}$)+(SAM (*x*_ij_−${\bar{x}}_{j}$))^2^+(SAM (${\bar{x}}_{j}$))^2^	213.199	2.995	0.040	0.410

### Interplay between blood stable isotopes and female mass, clutch initiation date, and total clutch mass

Female body mass, clutch initiation date, and total clutch mass were not significantly affected by either within‐individual or among‐individual effects of red blood cell δ^15^N or δ^13^C (all *F*_1_ ≤ 2.59; *P* ≥ 0.103). Overall, stable isotopes explained a low proportion of variance in models (0.1% to 7.3%; c.f. $R_{m}^{2}$ values in Table 4). In contrast, year and bird identity (included as random effects) contributed a much higher proportion to model fit in all models as reflected by the high $R_{c}^{2}$ values (59.7 to 85.1%; see Table 4).

**Table 4:** Structure of linear mixed effects models to test the effect of red blood cell δ^15^N and δ^13^C on female body mass, clutch initiation date, and total clutch mass. Each model contained either δ^15^N or δ^13^C as explanatory variable, with both the within‐individual‐centered data point (*x*_ij_−${\bar{x}}_{j}$) and the average value for each individual across years (${\bar{x}}_{j}$). All models further contained year and bird identity as independent random factors. AIC presents the Akaike information criterion. Marginal *R* ^2^ values ($R_{m}^{2}$) denote the variance explained only by fixed effects, whereas conditional *R* ^2^ values ($R_{c}^{2}$) express the variance explained by both fixed and random effects

	AIC	$R_{m}^{2}$	$R_{c}^{2}$
Female body mass			
δ^15^N (*x*_ij_−${\bar{x}}_{j}$)+δ^15^N (${\bar{x}}_{j}$)	225.912	0.066	0.851
δ^13^C (*x*_ij_−${\bar{x}}_{j}$)+δ^13^C (${\bar{x}}_{j}$)	225.510	0.073	0.851
Clutch initiation date			
δ^15^N (*x*_ij_−${\bar{x}}_{j}$)+δ^15^N (${\bar{x}}_{j}$)	326.230	0.001	0.597
δ^13^C (*x*_ij_−${\bar{x}}_{j}$)+δ^13^C (${\bar{x}}_{j}$)	321.755	0.056	0.605
Total clutch mass			
δ^15^N (*x*_ij_−${\bar{x}}_{j}$)+δ^15^N (${\bar{x}}_{j}$)	249.908	0.058	0.809
δ^13^C (*x*_ij_−${\bar{x}}_{j}$)+δ^13^C (${\bar{x}}_{j}$)	249.562	0.065	0.809

## Discussion

### Individual specialization across time

Red blood cell stable isotopes indicated a moderate degree of individual isotopic specialization, with the individuals' isotopic niches being 52% and 68% as broad as the population's niche, for δ^15^N and δ^13^C, respectively. Compared to the average documented WIC/TNW ratio in studies documenting individual specialization across taxa (0.66 ± 0.21 SD; Araújo et al. 2011), our results are therefore in the average to below‐average range, indicating significant individual specialization in foraging behavior during the prebreeding period. This also coincides with our result of significant repeatability of red blood cell δ^15^N and δ^13^C across years. While individual females therefore appeared consistent in their use of foraging areas and trophic level of prey during the prebreeding period across several years, we could not confirm these results for the premolt period. Feather δ^15^N and δ^13^C showed a high within‐individual variation and were not repeatable within individuals across years. SRP have so far been seen as food generalists with a high variability in their diet over space and time (reviewed in Pütz et al. 2013). Our current results suggest that our study population consists of isotopic specialists during the prebreeding period and of isotopic generalists before molt (Bearhop et al. 2004). Thus, female SRP switch within the course of a year between type A generalists (i.e., generalist individuals utilizing a wide range of food types/foraging areas) and type B generalists (i.e., individuals specializing on different food types/foraging areas) as defined by Bearhop et al. (2004). Such a seasonal alternation in specialization behavior is highly interesting and has rarely been described (but see Herrera et al. 2008; Hammerschlag et al. 2010). Most studies on individual isotopic specialization are based on a single sampling event (often obtaining multiple tissue samples per individual; e.g., Jaeger et al. 2010) or sampling multiple times within the course of only one breeding season or year (e.g., Ceia et al. 2012). Only few studies so far have taken data from the same individuals across multiple years into account (and these are usually restricted to only one time period/life‐history stage; reviewed in Araújo et al. 2011; but see Wakefield et al. 2015 for a recent multiyear study). Our results emphasize that the degree of specialization in foraging behavior may differ among time periods within the course of 1 year, even when being consistent within the same season across years. Therefore, we caution that the degree of individual specialization within a population may be highly time‐dependent. For future studies on individual specialization, multiple sampling events across different time periods within and among years would therefore be desirable.

From a life‐history point of view, it appears interesting that female SRP show different strategies in regard to isotopic specialization between the prebreeding and premolt periods. During both periods, birds need to obtain adequate body reserves for the subsequent fasting bouts and for the synthesis of eggs and feathers, respectively (see Pütz et al. 2013 for an overview of the annual life cycle). Successful foraging is therefore crucial during both periods to maximize reproductive investment (Dehnhard et al. 2015a) and – even more critical – avoid starvation during molt (Keymer et al. 2001). A potential explanation why SRP nevertheless show different strategies during prebreeding and premolt could be related to different time constraints. During winter, SRP are absent from the colonies for about 5 months, which allows them to disperse widely (Pütz et al. 2006; Thiebot et al. 2015). Differential individual preferences for certain wintering areas (and/or prey occurring there) inevitably affect the prebreeding isotopic compositions (see Dehnhard et al. 2011) and could result in high among‐individual variability in δ^15^N and δ^13^C (Thiebot et al. 2015). Thus, among‐individual differences in prebreeding foraging areas (and consistency therein across years; also see Wakefield et al. 2015) in combination with spatial differences in the availability of prey (or the preys' isotopic composition) are a likely explanation for the here‐found individual specialization in foraging behavior during the prebreeding period. In contrast, during the rather short time period between fledging of chicks and molt (approximately 3–7 weeks during which body reserves for molt are accumulated; Warham 1963; Strange 1982), adult females may not disperse as far or may utilize one specific foraging region (as observed by Thiebot et al. 2014 for the closely related macaroni penguin *Eudyptes chrysolophus*) with a dominating prey type. Both possibilities could explain the relatively small among‐individual isotopic variation in feathers compared to red blood cells from the prebreeding period. Our results of low among‐individual variation in feather δ^15^N and δ^13^C furthermore coincide with the low variation found in the same isotopic elements in (whole) blood of molting macaroni penguins (Thiebot et al. 2014). Importantly, red blood cells and feathers differ in their trophic fractionation (Bearhop et al. 2002; Cherel et al. 2005b), which likely explains the consistently higher δ^13^C values in feathers compared to red blood cells. However, this should not affect the total niche width when focusing on the tissues separately as we did. Also, we assume that fasting during molt and therefore the building of feathers from fat and protein stores as compared to the more direct assimilation of food resources for the formation of red blood cells did not affect our results regarding specialization. Although we did not sample blood and feathers simultaneously during the molt period, previous studies have shown comparable among‐individual variability in both tissues (Cherel et al. 2005a,b).

Individual differences in fractionation factors, for example, due to differences in individual physiology such as nutritional condition (Hobson et al. 1993) may – besides dietary specialization – also have a small effect on the TNW. However, such effects can be assumed to be rather small (Bearhop et al. 2004), and furthermore, one would expect to find the same individual effect on both feathers and red blood cells. Therefore, our differential results for the level of individual specialization from red blood cells and feathers should not be an artifact due to the different tissue types but truly reflect behavioral differences between the two different time periods.

### Effects of environmental variability on stable isotopes

Variations in red blood cell and feather δ^13^C and δ^15^N in SRP females were not explained by environmental variables included in the models, namely SAM, SOI, and local SSTA. Our selection of environmental variables was based on existing literature which showed that especially SAM and SSTA are linked to breeding phenology, body mass, and survival of SRP (Dehnhard et al. 2013b, 2015a,b).

Admittedly, interactions among factors of the environment and the food web are complex. Environmental conditions might not only affect the availability of different prey types but may also affect the winter distribution of seabirds differentially (Veit and Manne 2015). Moreover, we were not able to include information on the isotopic composition of prey species (i.e., the isotopic baseline and isoscape) in this study. We therefore cannot assess whether and how these isotopic values differed among years and over the broad spatial scale utilized by SRP during the prebreeding and premolt periods (Dehnhard et al. 2011; Ratcliffe et al. 2014). Annual and spatial variation in some prey species has been previously described by Quillfeldt et al. (2015), and this may have affected our results. Specifically, year differences in the isotopic baseline could have precluded us from finding a relationship between environmental conditions and the penguins' isotopic values. Similarly, the differences in penguin stable isotopes among years may be related rather to differences in the isotopic baseline than to true interannual differences in diet or foraging areas. The lack of baseline isotopic values therefore leads to some uncertainty about the interpretation of our data. Nevertheless, this should not have affected our results about the isotopic specialization of individuals. Furthermore, for testing the relationship between stable isotopes and female body mass and breeding behavior, we corrected for a potential baseline effect by standardizing both isotope values as well as female body mass and breeding parameters. These results are therefore independent of potential isotopic baseline effects.

### Effects of isotopes on breeding behavior

Against our expectation, we found no among‐individual effect of isotopes on either female body mass, clutch initiation date, or total clutch mass. Thus, the earlier described consistency of individuals in their prebreeding body mass and egg mass (Dehnhard et al. 2015a) was independent of the specialization of individuals on their prebreeding foraging areas or trophic level of prey. While relationships between individual specialization in foraging behavior and the timing of breeding (Anderson et al. 2009) and reproductive success (Spear 1993; Annett and Pierotti 1999; Patrick and Weimerskirch 2014) have been described previously, implications of individual specialization on the adults' mass, egg mass, or egg volume appear rather rare (but see Annett and Pierotti 1999; Votier et al. 2004, 2010; Masello et al. 2013). Furthermore – and in agreement with our results – several other studies could not find a connection between specialization in foraging behavior and adult body mass index (Ceia et al. 2012), food delivery rates (Watanuki et al. 2010), weaning mass (Ducatez et al. 2008), fledging success (Votier et al. 2004; Woo et al. 2008), and long‐term survival (Woo et al. 2008; van de Pol et al. 2010). The adaptive significance of individual specialization in foraging behavior in SRP therefore remains unclear. It might simply reflect fidelity to foraging sites or diet, independent of intraspecific competition (Baylis et al. 2015). Alternatively, the here‐observed pattern of individual specialization in foraging behavior may reduce intraspecific competition without being coupled to specific individual advantages. Finally, adaptive benefits may only occur under certain conditions or in certain years, with effects leveling out in the long term (see Woo et al. 2008; van de Pol et al. 2010).

Along with the lack of among‐individual effects, we could also find no within‐individual effect of either δ^13^C or δ^15^N on female body mass or breeding behavior. Consequently, there was no indication of phenotypic plasticity in female body mass or breeding behavior in response to changes in diet or foraging behavior.

## Conclusion

Individual specialization in foraging behavior may have important consequences on evolutionary adaptations when counteracting phenotypic plasticity. Previously considered a generalist species, female SRP show consistent among‐individual differences in body mass and egg masses across years, and this was suggested to be related to individual specialization in foraging behavior. We investigated variation in stable isotopic compositions both within and among individuals and the effects of these variations on female body mass, the timing of breeding, and the investment into breeding across multiple years in this species. Our findings emphasized that the degree of individual isotopic specialization within generalist populations may vary, so that a population may be composed of isotopic specialists at one time period but consists of true generalists during another period of the annual cycle. Animals commonly change their diets in the course of a year – for example, to cope with specific demands during breeding or migration (Parrish 1997; van Gils et al. 2005) – but a shift from isotopic specialization to isotopic generalization has rarely been described in the literature. Importantly, mention of specialists and generalists should therefore also take the time period into consideration, as the behavior might change over the course of the year.

SRP females showed significant individual specialization in δ^15^N and δ^13^C only during the prebreeding period, and these among‐individual differences remained consistent across years. Surprisingly, though, and contrary to the previous suggestion, this individual specialization in foraging behavior was not related to individual consistency in body mass or investment into egg mass. Variation in isotopic values was also unrelated in any form to the studied environmental variables (SAM, SOI, and local SSTA). Consequently, we were unable to confirm any phenotypic plasticity in the isotopic composition in response to environmental variables. Environmental variability, at least for our 7‐year dataset, appears not to counteract individual isotopic specialization. With the long‐term “experiment” of ongoing global climate change, our data provide a useful comparison to studies in future decades to give a definite answer on whether increasing environmental variability will counteract individual long‐term specialization or not.

## Data Accessibility

Data will be made available on Dryad after acceptance of the manuscript.

## Conflict of Interest

None declared.

## Supporting information


**Figure S1.** Stable nitrogen (panel a) and carbon (panel b) isotopes of 48 different females that were sampled during the early breeding season 2009/10 while fasting.
