# Supplementary material for: Is individual consistency in body mass and reproductive decisions linked to individual specialization in foraging behavior in a long‐lived seabird?
Source: Ecol Evol. 2016 Jun 8;6(13):4488–501. doi: 10.1002/ece3.2213 (PMC4930996; doi:10.1002/ece3.2213)
Supplement: Supplementary file 1 — Figure S1. Stable nitrogen (panel a) and carbon (panel b) isotopes of 48 different females that were sampled during the early breeding season 2009/10 while fasting. [file ECE3-6-4488-s001.docx]

***Supplement 1. Correction of body mass and stable isotope data for birds not sampled at their clutch initiation date***

For females that were not captured on their clutch initiation date (N = 16 out of 130 records), we applied corrections for body mass and stable isotope data since fasting has an effect on both body mass and blood *δ*^15^N (Cherel *et al.* 2005).

For body mass, we applied the same correction factor as previously published in Dehnhard et al. (2015). Thus, we assumed a linear body mass decrease by 33.3 g per fasting day and used this value to back-calculate body mass at clutch initiation.

For stable isotopes, we used data from 48 different females that were sampled throughout the fasting period in 2009. First sampling (N = 16 birds) occurred shortly (approximately 1–3 days) after arrival of females in the colony. Another 16 birds were sampled 24 days later (during the first incubation shift) and 16 more birds were sampled during the second incubation shift (36–38 days after initial sampling). We performed a linear model with fasting time (in days since first capture) as only explanatory variable, separately for *δ*^15^N and *δ*^13^C as dependent variable. *δ*^15^N increased significantly with time (F_1_ = 4.77, P = 0.034), while there was no significant effect of fasting on *δ*^13^C (F_1_ = 0.34, P = 0.563) (see Fig. S1). We therefore only extracted the estimate from the model for *δ*^15^N (f(x) = 13.095 + 0.026*x) and consequently assumed a linear increase by 0.026 ‰ *δ*^15^N per day. We used this value as a correction factor for birds that were not captured on clutch initiation date. Notably, we found a very similar effect size for fasting on red blood cells as previously presented by Cherel et al. (2005) from an individual-based study on king penguins (*Aptenodytes patagonicus*).

Fig. S1. Stable nitrogen (panel a) and carbon (panel b) isotopes of 48 different females that were sampled during the early breeding season 2009/10 while fasting. 16 birds were sampled shortly after arrival in the colony (day 0), another 16 birds 24 days later (during the first incubation shift) and 16 further birds were sampled during the second incubation shift (36–38 days after initial sampling). The regression line reflects the significant increase in δ^15^N as a result of fasting.

**References**

Cherel, Y., Hobson, K.A., Bailleul, F. & Groscolas, R. (2005) Nutrition, physiology, and stable isotopes: new information from fasting and molting penguins. *Ecology,* **86,** 2881-2888.

Dehnhard, N., Eens, M., Demongin, L., Quillfeldt, P. & Poisbleau, M. (2015) Individual consistency and phenotypic plasticity in rockhopper penguins: female but not male body mass links environmental conditions to reproductive investment. *PLoS ONE,* **10,** e0128776.
